# Draft Genome Sequence of *Mediterraneibacter* sp. nov. Strain gm002, Isolated from Human Feces

**DOI:** 10.1128/MRA.00217-19

**Published:** 2019-08-01

**Authors:** Chien-Hsun Huang, Jong-Shian Liou, Chun-Lin Wang, Lina Huang

**Affiliations:** a Bioresource Collection and Research Center, Food Industry Research and Development Institute, Hsinchu, Taiwan, Republic of China; University of Maryland School of Medicine

## Abstract

Here, we report a draft genome sequence of *Mediterraneibacter* sp. strain gm002, isolated from human feces in Taiwan. This strain represents a novel species according to whole-genome-based comparisons using the average nucleotide identity (ANI) and digital DNA-DNA hybridization (dDDH) thresholds. The genome assembly comprised 3,568,768 bp, with a 38.7% G+C content.

## ANNOUNCEMENT

During a study aimed at the isolation of novel bacteria present in the human gut, we isolated *Mediterraneibacter* sp. strain gm002 from a fecal sample from a healthy Taiwanese man in Taiwan, using a culturomics approach (1). Based on sequencing of the full-length 16S rRNA gene, strain gm002 was found to exhibit 97.9% sequence identity with Mediterraneibacter lactaris ATCC 29176^T^ (GenBank accession number GCA_000155205), indicating that this isolate may represent a new species.

Mediterraneibacter sp. gm002 was grown on modified Gifu anaerobic medium agar (Nissui Pharmaceutical Co., Tokyo, Japan) at 37°C for 2 days under anaerobic conditions. Genomic DNA was extracted from strain gm002 using an EasyPrep HY genomic DNA extraction kit (Tools, Taiwan). The libraries were constructed using the NEBNext DNA library prep mastermix kit (NEB, Inc.), and the DNA was sequenced on an Illumina HiSeq 4000 platform using 2 × 150-bp paired-end reads at the Beijing Novogene Bioinformatics Technology Co., Ltd. (Beijing, China). Sequencing of strain gm002 generated 8,868,158 paired-end reads. The reads with low-quality (*Q* value of ≤38) nucleotides exceeding 40 bp, nontemplated nucleotides (N-nucleotides) exceeding 10 bp, or a read overlap with the adapter exceeding 15 bp were removed using the quality control pipeline by Novogene Bioinformatics Technology Co., Ltd. After adapter filtering and quality trimming, there were 7,720,936 clean reads remaining. *De novo* assembly was performed using the clean reads with SOAPdenovo software version 2.04 using the default settings (2), which resulted in 69 scaffolds (>500 bp; *N*_50_, 116,634 bp) with an average coverage of 270×, comprising a total of 3,568,768 bp with a G+C content of 38.7%. Genome annotation was completed by submission to the NCBI Prokaryotic Genome Automatic Annotation Pipeline (PGAAP) to obtain a total of 3,370 predicted genes, 11 rRNAs, 66 tRNAs, and 4 noncoding RNAs (3). Moreover, annotation of the protein-coding genes with Rapid Annotations using Subsystems Technology (RAST) version 2.01 (4) predicted 3,399 protein-coding genes, 1,837 of which had functional categories of SEED subsystems, while analysis with antiSMASH version 4.2 (5) predicted 11 biosynthetic gene clusters, which include biosynthetic gene clusters for exopolysaccharide, pradimicin, polysaccharide, equibactin, and emulsan.

In addition, the average nucleotide identity (ANI) value was estimated using Orthologous ANI (OrthoANI) (6), and digital DNA-DNA hybridization (dDDH) was performed using the Genome-to-Genome Distance Calculator (GGDC) with the recommended formula 2 (7). The ANI and dDDH values between strain gm002 and type strains of *M. lactaris* ATCC 29176^T^ were 74.6% and 24.6%, respectively. Both values are below assigned thresholds used for species delineation and revealed that strain gm002 represents a novel species.

### Data availability.

This whole-genome shotgun project has been deposited at DDBJ/ENA/GenBank under the accession number SIHS00000000 (BioProject number PRJNA522945). The version described in this paper is the first version, SIHS01000000. Raw sequences were deposited in the NCBI SRA database under accession number SRR8894172.
